# Changes in Reactivity *In Vitro* of CD4^+^CD25^+^ and CD4^+^CD25^−^ T Cell Subsets in Transplant Tolerance

**DOI:** 10.3389/fimmu.2017.00994

**Published:** 2017-08-22

**Authors:** Bruce M. Hall, Catherine M. Robinson, Karren M. Plain, Nirupama D. Verma, Giang T. Tran, Masaru Nomura, Nicole Carter, Rochelle Boyd, Suzanne J. Hodgkinson

**Affiliations:** ^1^Immune Tolerance Laboratory, Department of Medicine, Ingham Institute, University of New South Wales, Liverpool Hospital, Liverpool, NSW, Australia; ^2^Faculty of Veterinary Sciences, University of Sydney, Cobbity, NSW, Australia; ^3^Department of Surgery, Nakashibetsu Hospital Shibetu-gun Nakashibetsu-cho, Hokkaido, Japan; ^4^Faculty of Medicine and Health Sciences, Macquarie University, Macquarie Park, NSW, Australia

**Keywords:** CD4^+^ T cells, CD4^+^CD25^+^ T cells, Treg, antigen-specific Treg, transplantation, tolerance

## Abstract

Transplant tolerance induced in adult animals is mediated by alloantigen-specific CD4^+^CD25^+^ T cells, yet in many models, proliferation of CD4^+^ T cells from hosts tolerant to specific-alloantigen *in vitro* is not impaired. To identify changes that may diagnose tolerance, changes in the patterns of proliferation of CD4^+^, CD4^+^CD25^+^, and CD4^+^CD25^−^ T cells from DA rats tolerant to Piebald Virol Glaxo rat strain (PVG) cardiac allografts and from naïve DA rats were examined. Proliferation of CD4^+^ T cells from both naïve and tolerant hosts was similar to both PVG and Lewis stimulator cells. In mixed lymphocyte culture to PVG, proliferation of naïve CD4^+^CD25^−^ T cells was greater than naïve CD4^+^ T cells. In contrast, proliferation of CD4^+^CD25^−^ T cells from tolerant hosts to specific-donor PVG was not greater than CD4^+^ T cells, whereas their response to Lewis and self-DA was greater than CD4^+^ T cells. Paradoxically, CD4^+^CD25^+^ T cells from tolerant hosts did not proliferate to PVG, but did to Lewis, whereas naïve CD4^+^CD25^+^ T cells proliferate to both PVG and Lewis but not to self-DA. CD4^+^CD25^+^ T cells from tolerant, but not naïve hosts, expressed receptors for interferon (IFN)-γ and IL-5 and these cytokines promoted their proliferation to specific-alloantigen PVG but not to Lewis or self-DA. We identified several differences in the patterns of proliferation to specific-donor alloantigen between cells from tolerant and naïve hosts. Most relevant is that CD4^+^CD25^+^ T cells from tolerant hosts failed to proliferate or suppress to specific donor in the absence of either IFN-γ or IL-5. The proliferation to third-party and self of each cell population from tolerant and naïve hosts was similar and not affected by IFN-γ or IL-5. Our findings suggest CD4^+^CD25^+^ T cells that mediate transplant tolerance depend on IFN−γ or IL-5 from alloactivated Th1 and Th2 cells.

## Introduction

Tolerance to transplanted tissue is alloantigen-specific, as second grafts from the same donor strain are accepted whereas third-party grafts are rejected (1–3). Classical transplant tolerance is induced by infusion of donor cells in neonatal rodents (1) or in adults depleted of lymphoid cells (4, 5). These protocols aim for clonal deletion with reduced or absent proliferation of CD4^+^ T cells in mixed lymphocyte culture (MLC) to specific-donor, but not to third-party. However, MLC and cell-mediated lysis assays have a poor predictive value for tolerance in neonatal tolerance (6) and human renal transplants (7, 8).

In murine models, specific transplant tolerance can be induced by blocking the initial immune response (2, 9, 10). Donor cell infusions and lympho-hemopoietic chimerism are not required, neither is depletion of T cells. Alloreactive CD4^+^ and CD8^+^ T cells are activated and infiltrate the graft during tolerance induction (11–13). After months, without further immunosuppression, animals accept a second graft from donor strain but reject third-party grafts (14). Peripheral lymphocytes from these tolerant hosts respond to specific-donor in MLC (14), contain donor-specific cytotoxic T cells (14), and react to specific-donor in graft-vs-host assays (15). This “operational” or “split tolerance” (2) is dependent for its induction and maintenance on alloantigen-specific CD4^+^ T regulatory cells (Treg) (3, 10, 16, 17) especially CD4^+^CD25^+^ Treg (18–20). These antigen-specific Treg prevent host effector cells mediating rejection (3, 18, 19) and can convert host effector cells to Treg (17). CD4^+^CD25^−^ T cells from tolerant animals are not clonally deleted and effect rejection of specific-donor grafts in adoptive hosts (10, 16, 17).

While CD4^+^ T cells from tolerant hosts transfer tolerance to an immunodeficient host and can suppress rejection mediated by small numbers of naïve CD4^+^ T cells. Paradoxically, *in vitro* CD4^+^ T cells from tolerant hosts have a normal response in MLC to specific donor and third-party alloantigen. Thus, suppressor assays are not feasible.

Antigen-specific CD4^+^CD25^+^ T cells from tolerant hosts express forkhead box P3 (FOXP3), but are different to naïve CD4^+^CD25^+^FOXP3^+^ Treg (tTreg) derived from the thymus. Although naïve tTreg (21) can induce transplant tolerance, maintenance of tolerance requires activated antigen-specific Treg (22).

There are two findings that underpin the hypothesis of this study. First, CD4^+^ T cells from tolerant hosts lose their capacity to transfer transplant tolerance when cultured in MLC with donor alloantigen, as the surviving CD4^+^ T cells effect specific-donor rejection (16, 18, 23, 24). However, culture of CD4^+^ T cells from tolerant hosts in cytokine-rich supernatant from Concanavalin A (ConA) activated spleen cells, together with specific-donor stimulator cells, promotes survival of CD4^+^ T cells with the capacity to transfer tolerance (23, 24). IL-2 alone (23) or IL-4 alone (24) do not sustain tolerance transferring CD4^+^ T cells.

Second, naïve tTreg cultured with alloantigen and IL-2 are induced to express receptors for other Th1 cytokines interferon (IFN)-γ (IFNGR) (22) and IL-12 (IL-12Rβ2) (25) but do not express IL-5Rα. tTreg cultured with specific-alloantigen and IL-4 express specific receptor for the Th2 cytokine IL-5 (IL-5Rα) (22, 26) and do not express IFNGR or IL-12Rβ2. These alloantigen-specific Treg have increased potency to suppress specific donor allograft rejection (22, 25). Thus, our hypothesis was that antigen-specific Treg in tolerant hosts need stimulation by specific-alloantigen and either IFN-γ or IL-5 (26, 27).

Here, we examined patterns of proliferation of CD4^+^, CD4^+^CD25^+^, and CD4^+^CD25^−^ T cells from naïve and tolerant host in MLC with stimulator cells from the tolerated alloantigen, third-party alloantigen, or self. We were looked for differences in patterns of response by cells from tolerant and naïve rats that may indicate alloantigen-specific tolerance.

Four key differences were observed: first, CD4^+^CD25^+^ T cells from tolerant hosts did not inhibit proliferation of CD4^+^CD25^−^ T cell from tolerant hosts to specific-donor but did inhibit responses to third-party in MLC, whereas naïve CD4^+^CD25^+^ T cells inhibited naïve CD4^+^CD25^−^ T cell proliferation to all alloantigens in MLC. Second, CD4^+^CD25^+^ T cells from tolerant hosts did not proliferate to specific-donor alloantigen but did to third-party, whereas naïve CD4^+^CD25^+^ T cells proliferated to all alloantigens. Third, CD4^+^CD25^+^ T cells from tolerant hosts but not from naïve hosts expressed receptors for IFN-γ and IL-5. Fourth, addition of either IFN-γ or IL-5 promoted proliferation of CD4^+^CD25^+^ T cells from tolerant hosts, but not naïve CD4^+^CD25^+^ T cells, to specific-donor but not to third-party alloantigen.

## Materials and Methods

### Animals

DA (RT1^a^), Piebald Virol Glaxo rat strain (PVG) (RT1^c^), and Lewis (RT-1^l^) rats were bred and maintained in the animal house, Liverpool Hospital. All animals were fed standard chow and given water *ad libitum*. The study was carried out in accordance of the “Australian Code for the Care and Use of Animals for Scientific Purposes (NHMRC)” and Animal Ethics Committee of the University of New South Wales (UNSW), Australia. Animal experimental protocols were approved by the Animal Ethics Committee of the UNSW Australia.

### Operative Procedures

DA rats weighing 180–230 g were anesthetized with either ether or isoflurane and heterotopically grafted with adult PVG heart (14). Graft rejection was assessed as cessation of palpable beat (21). Tolerance was induced by intraperitoneal injection of 7 mgm/kg of an anti-CD3 mAb (G4.18), as described (13, 14, 19). Hosts with good functioning grafts for >150 days were considered tolerant. The cells studied were from spleen and lymph nodes of tolerant animals, >150 days after transplantation (14, 19).

### mAb and Immunostaining

Anti-rat mAb used were G4.18 (CD3), Ox35 (CD4), MRCOx8 (CD8), MRCOx39 (CD25, IL-2R alpha chain), LECAM-1 (CD62L, l-selectin), and MRCOx33 (CD45RA, B cells) (BD-Pharmingen, San Diego, CA, USA). Anti-mouse/rat FOXP3 (FJK-16s) (eBioscience, San Diego, CA, USA) was used as per the manufacturer’s instructions. Immunostained lymphocytes were analyzed on a FACScan (Becton Dickenson, San Jose, CA, USA) using CellQuest software (Becton Dickenson).

### Cytokines

Recombinant (r) IL-2, rIL-4, rIL-5, rIL-10, rIL-12p70, rIL-13, rIFN-γ, and rTGF-β cytokines were produced and quantified, as described (22). Each cytokine was added to cultures at ≥200 U/ml. The IL-4-transfected cell line (28) was a gift of Dr. Barclay (Pathology, Oxford, UK).

### Cell Preparation and CD4^+^ T Cells Subset Separation

Single cell suspensions from spleen and lymph node were prepared and RBC lysed as described (29). An indirect panning technique to deplete CD8^+^ T and B cells, followed by CD25 enrichment using PE conjugated MRCOx39 mAb and anti-PE microbeads (Miltenyi Biotech Australia, Macquarie Park, NSW, Australia) as described (21, 25, 29). Enriched CD4^+^CD25^−^ T cells were >96% CD4^+^ with <3% CD25^hi^ cells. Enriched CD4^+^CD25^+^ T cells were 85–95% CD25^+^ with greatest enrichment for CD25^hi^ cells and had 70–80% FOXP3^+^cells.

### Mixed Lymphocyte Cultures

The methods were as previously described (22, 25, 29). Briefly, stimulator cells were irradiated cells from thymus of naïve rats, which do not produce T cell cytokines (13). Stimulator cells from thymus are as effective or more effective than irradiated spleen cells as stimulator cells (29). Stimulation in MLC with specific-donor PVG cells was compared to stimulation to self-DA and third party Lewis.

Micro-cultures in *U*-bottom microtiter plates (Greiner) contained 2 × 10^4^ stimulator cells and 2 × 10^5^ responder cells in a total volume of 200 µl. Proliferation was assessed by ^3^H thymidine incorporation, as described (22, 29). Each experimental sample had 3–6 replicates and results were expressed as a mean ± SD.

As naïve CD4^+^CD25^+^ T cells are poor at proliferation in MLC without IL-2, the methods were refined to eliminate non-specific background proliferation (22, 29). These modifications included use of 20% Lewis rat serum rather than fetal calf serum and irradiated thymic stimulator cells.

In time course assays, proliferation was assessed at days 2, 3, 4, 5, and 6. For limiting dilution assays, serial twofold dilutions of T cells subsets starting at 1 or 2 × 10^5^ cells/well were cultured with 2 × 10^4^ stimulators cells/well and were assayed for proliferation at day 4. Stimulation index was calculated as proliferation of test MLC with cytokine added divided by proliferation of the control MLC with no cytokine.

### Reverse Transcription Polymerase Chain Reaction (RT-PCR)

mRNA from cells was extracted and reverse transcribed as described (22, 25). Primers for *il-5ra, ifngr, il5, ifng, foxp3*, and methods of RT-PCR were as described (22, 25).

### Statistical Analyses

Parametric data were expressed as mean ± SD and were compared with Student’s *t*-test using Statview for Mac (Abacus Concepts, Berkley, CA, USA). Wilcoxon Signed Rank test was used for non-parametric results. Statistical significance was set at *p* < 0.05.

## Results

### Comparison of Immunostaining of Peripheral Lymphoid Cells from Naïve DA Rats and DA Rats Tolerant to a PVG Heart Allograft

Tolerance was induced by treatment of DA rats with anti-CD3 mAb (G4.18) at the time of transplantation with a heterotopic PVG heart graft, as described (13, 14, 19). Spleen and lymph node cells from tolerant hosts were from rats with good functioning heart grafts for over 150 days that were receiving no ongoing immunosuppressive therapy.

**Figure 1:**
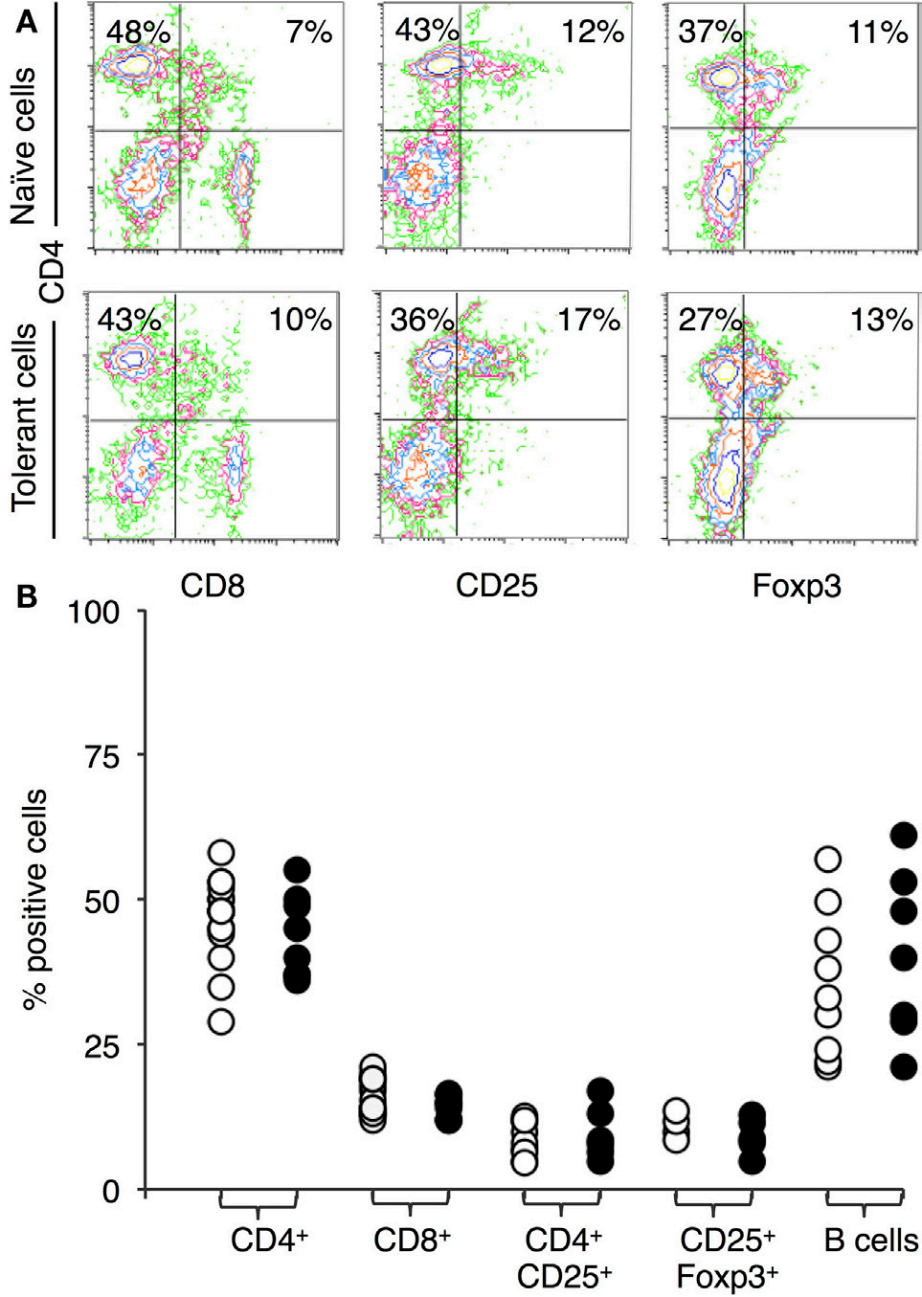
Immunostaining of peripheral lymphocytes: comparison of cells from DA rats tolerant to a Piebald Virol Glaxo rat strain (PVG) allograft and naïve DA. **(A)** FACS profiles of lymphocytes from naïve DA rats (upper panel) and DA rats tolerant to PVG heart graft (lower panel). *y*-axis represents CD4 and *x*-axis CD8 (left panel), CD25 (middle panel), and forkhead box P3 (FOXP3) (right panel). **(B)** Percentage of cells expressing a given cell surface molecule in naïve (○) and tolerant (●) animals, results from 4–16 different sets of animals. There was no significant difference in the proportion of CD4^+^ or CD8^+^ T cells showing anti-CD3 therapy did not deplete T cells. There was no increase in B cells with tolerance. The proportion of CD4^+^CD25^+^ T cells or CD25^+^FOXP3^+^ T cells was not increased in tolerant animals.

Figure 1A shows representative immunostaining and FACS analysis for CD4, CD8, CD25, and FOXP3. The proportion of lymphocyte subsets in lymph node and spleen of tolerant and naïve DA rats was similar for CD4^+^ T cells, CD8^+^ T cells, or B cells (Figure 1B). There was no increase in the proportion of CD4^+^CD25^+^ T cells in tolerant hosts, which remained at <20% of peripheral lymphocytes (Figure 1B, *n* = 16). 70–80% of CD4^+^CD25^+^ T cells expressed FOXP3 in both naïve and tolerant hosts.

### Proliferation in MLC of Naïve Unfractionated, CD4^+^, CD4^+^CD25^−^, and CD4^+^CD25^−^ T Cells

**Figure 2:**
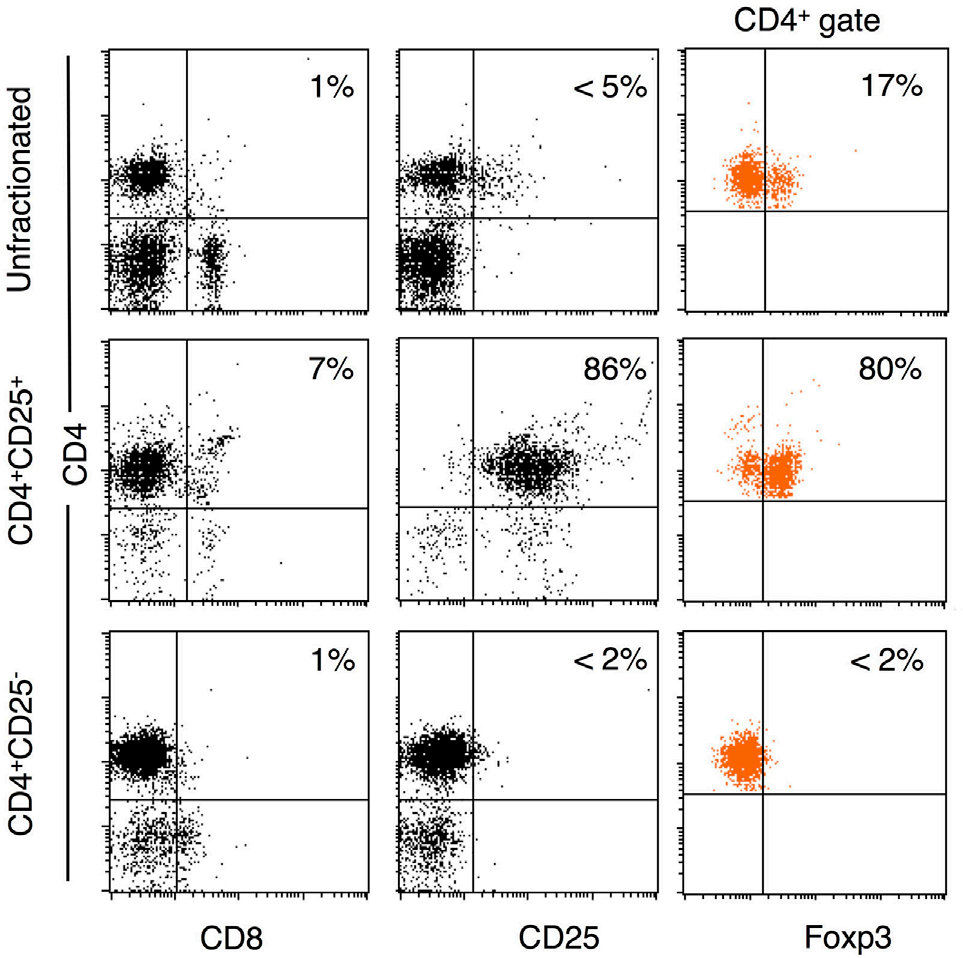
Enrichment of CD4^+^, CD4^+^CD25^−^, and CD4^+^CD25^+^ T cells from DA rats tolerant to Piebald Virol Glaxo rat strain (PVG) allograft. Lymphocytes from DA rats tolerant to PVG cardiac allograft were enriched for CD4^+^CD25^+^ T cells as in Section “Materials and Methods.” FACS profiles of unfractionated spleen and lymph node cells (top panel), and CD4^+^CD25^+^ (middle panel), and CD4^+^CD25^−^ (bottom panel) subpopulations. CD4^+^CD25^+^ T cell preparations were >85% CD25^+^ (middle left plot) and 70–80% expressed forkhead box P3 (FOXP3) (middle right plot). CD4^+^CD25^−^ T cell population had very few CD25^hi^ (bottom middle plot, <2%) or FOXP3^+^ (bottom right plot, <2%) cells.

The unfractionated, CD4^+^, CD4^+^CD25^+^, and CD4^+^CD25^−^ populations were prepared as described in the Section “Materials and Methods.” Figure 2 shows a representative FACS analysis of enriched T cell subsets from tolerant hosts. CD4^+^CD25^−^ T cells had <1% CD8^+^, <2% CD25^hi^, and <2% FOXP3^hi^. CD4^+^CD25^+^ T cells were >85% CD25^+^ and 70–80% FOXP3^+^ (Figure 2). This subpopulation had 5–10% of CD8^lo^T cells that was also CD4^+^. Preparations had 1–5% B cells, but these cells do not proliferate in rat MLC (30).

**Figure 3:**
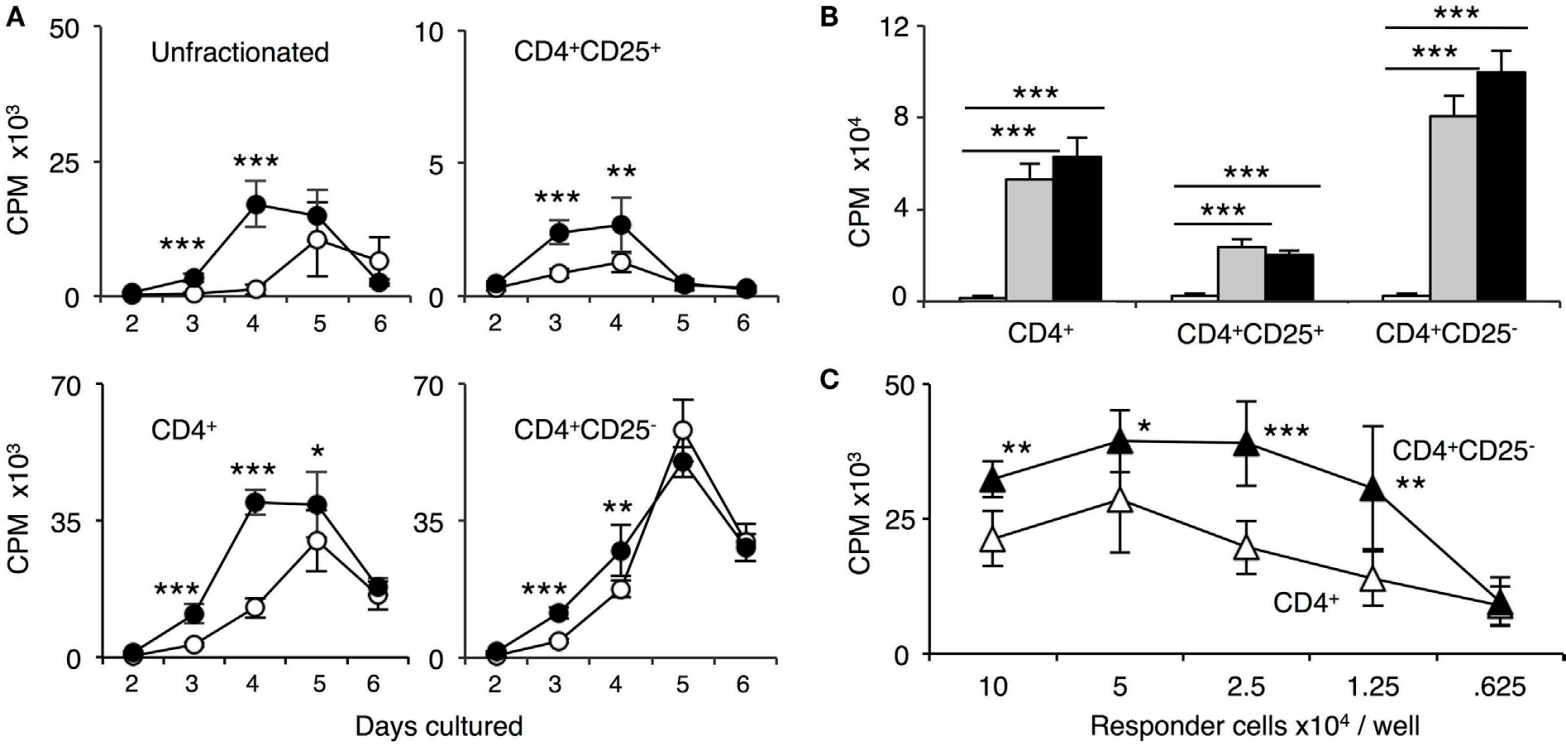
Comparison of proliferation of naïve unfractionated, CD4^+^ T cells and CD4^+^ T cell subsets in mixed lymphocyte culture (MLC) to self-DA, and allogeneic stimulators Piebald Virol Glaxo rat strain (PVG) and Lewis. Proliferation of unfractionated and CD4^+^, CD4^+^CD25^−^, and CD4^+^CD25^+^ T cells from naïve DA rats in MLC was assessed. The response to fully allogeneic PVG stimulator cells was compared to self-DA **(A,B)** and other fully allogeneic Lewis stimulator cells **(B)**. Significant differences are shown as *p < 0.05, **p < 0.01, and ***p < 0.001. **(A)** Time course with unfractionated cells and CD4^+^ T cells showed peak proliferation to PVG (●) at day 4 and maintained at day 5, after which it waned. There was significant proliferation to DA (○), peaking at day 5. With CD4^+^CD25^−^ T cells, proliferation was greatest at day 4 and day 5; however, there was little difference in response to PVG and self-DA. Proliferation of CD4^+^CD25^−^ T cells was greater than unfractionated and enriched CD4^+^ T cells. CD4^+^CD25^+^ T cells proliferation was much less than that of CD4^+^ or CD4^+^CD25^−^ T cells. There was a detectable response to PVG at days 3 and 4 that waned by day 5 and 6. In subsequent experiments, proliferation of CD4^+^CD25^+^ T cells was assayed at day 4, and of CD4^+^ and CD4^+^CD25^−^ at day 4 or 5. **(B)** Comparison of proliferation in MLC of CD4^+^ T cells and subsets to self-DA, PVG, and Lewis stimulator cells. All populations had similar responses to PVG (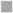) and Lewis (◼) that were greater than proliferation to self-DA (◻) (***p < 0.001). **(C)** Comparison of responses of serially diluted naïve DA CD4^+^ T cells (△) and CD4^+^CD25^−^ T cells (▲) in MLC stimulated by a constant number of PVG cells. CD4^+^CD25^−^ T cells had significantly higher proliferation to PVG compared to CD4^+^ T cells indicating that removal of naïve CD4^+^CD25^+^ T cells significantly enhanced proliferation.

The time course of proliferation of naïve DA cells to self-DA and fully allogeneic PVG is illustrated in Figure 3A. Naïve unfractionated and enriched CD4^+^ T cells had a similar time course with proliferation to PVG peaking at day 4 and 5 and waning by day 6. The response to self-DA was delayed and peaked at day 5.

With naïve CD4^+^CD25^−^ T cells, the proliferation to PVG and to DA was similar at the peak on day 5. Prior to that, there was a slightly greater proliferation to PVG compared to self-DA. In subsequent experiments with CD4^+^ and CD4^+^CD25^−^ T cells, proliferation was assayed at day 4, when the differences between response to PVG and DA were greatest.

Naïve CD4^+^CD25^+^ T cell proliferation was much less than unfractionated lymphoid cells, CD4^+^ T cells, or CD4^+^CD25^−^ T cells (Figures 3A,B). Proliferation to PVG was significantly greater than to self-DA at day 3 and 4, after which it waned. Counts were small and never more than two thousand, often in the hundreds. In subsequent experiments, CD4^+^CD25^+^ T cell proliferation was assayed at day 3 or 4.

### Comparison of the Response of CD4^+^ T Cells from Tolerant and Naïve Hosts in MLC

**Figure 4:**
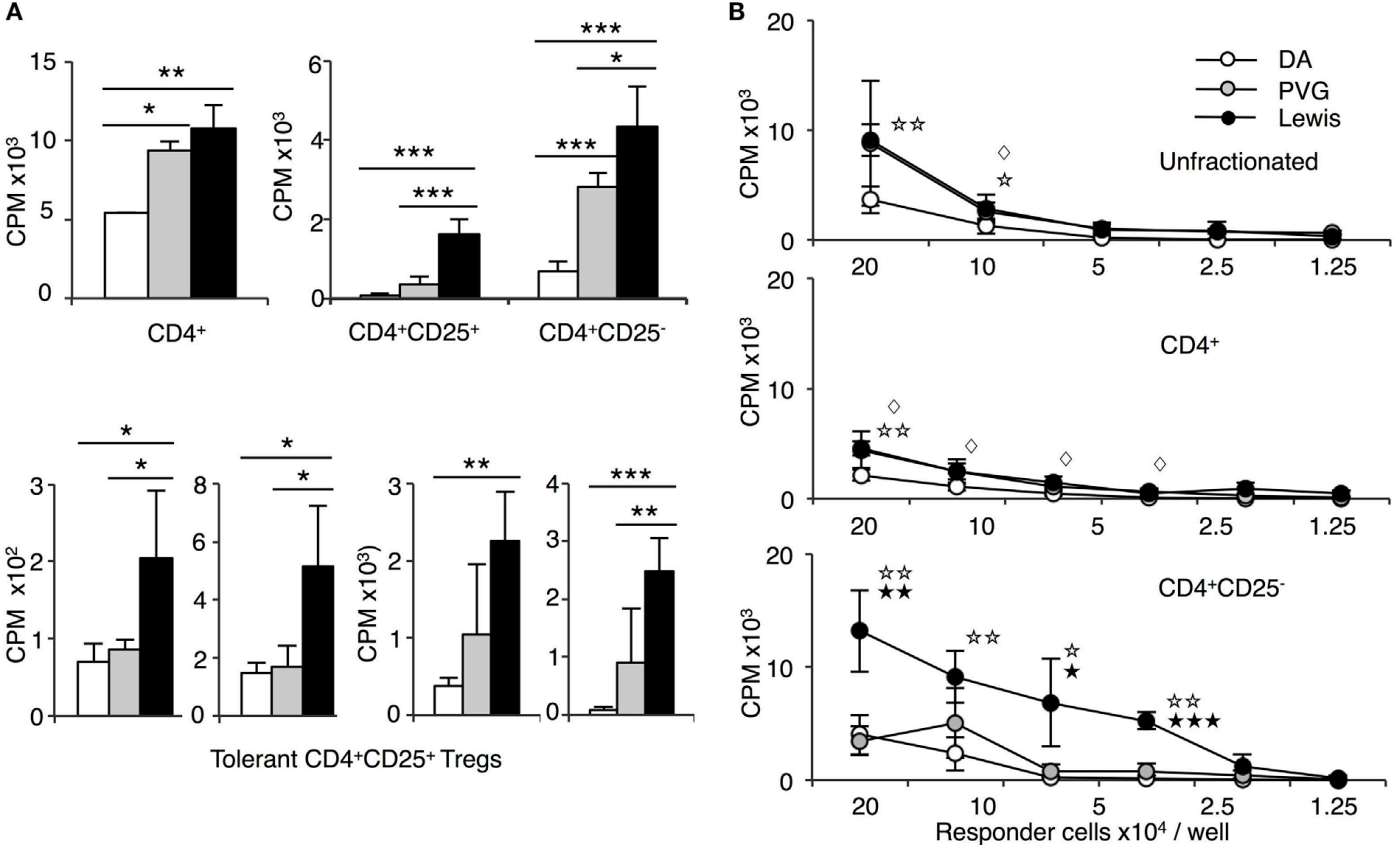
Comparison of proliferation of CD4^+^, CD4^+^CD25^−^, and CD4^+^CD25^+^ T cells from tolerant hosts in mixed lymphocyte culture (MLC) to self-DA, specific-donor Piebald Virol Glaxo rat strain (PVG), and to third-party Lewis. Proliferation of CD4^+^, CD4^+^CD25^−^, and CD4^+^CD25^+^ T cells subjected to MLC with either self (DA), specific-donor (PVG), or third party (Lewis) at day 4, assessed by ^3^H thymidine incorporation and expressed as cpm. **(A)** Proliferation of cell subsets to donor antigens in MLC. CD4^+^ T cells from tolerant hosts (top left panel) had a similar response to specific-donor PVG (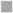) and third-party Lewis (◼) that was much greater than to self-DA (◻). Two other experiments had similar results. For CD4^+^CD25^−^ T cells from tolerant hosts (top right panel), the response to specific-donor PVG was less than to third-party Lewis. This response of CD4^+^CD25^−^ T cells was not greater than that of CD4^+^ T cells. CD4^+^CD25^+^ T cells from DA rats tolerant to PVG graft did not respond to specific-donor PVG, and this response was not greater than to self-DA (top right panel). CD4^+^CD25^+^ T cells from tolerant hosts had significantly higher proliferation to third-party Lewis compared to specific-donor PVG or self-DA. Bottom row panel shows 4 of the 8 replicate experiments that confirmed CD4^+^CD25^+^ T cells from tolerant hosts did not respond or had low response to specific-donor strain PVG compared to their response to third-party Lewis. Significant differences in responses are marked as * if *p* < 0.05; ** if *p* < 0.01, and *** if *p* < 0.001. **(B)** Limiting dilution assay of cells from tolerant hosts in MLC. The response of unfractionated cells (top panel) and CD4^+^ T cells (middle panel) was similar to specific-donor PVG (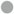) and third party Lewis (●) at all points, and was greater than that to self-DA (○). CD4^+^CD25^−^ T cells from tolerant hosts (bottom panel), in contrast to CD4^+^ T cells (middle panel), had a marked increased response to third-party Lewis but not to specific-donor PVG. Significant at three dilutions. Collectively, these findings suggest that CD4^+^CD25^+^ T cells from tolerant animals paradoxically did not suppress the response of CD4^+^CD25^−^ T cells from tolerant hosts to specific-donor alloantigen, but retain the capacity to suppress responses to self-DA cells and third-party Lewis alloantigen. Symbols for significant differences are: ⋄ PVG vs DA; ☆ Lewis vs DA; ★ Lew vs PVG; 1 symbol if *p* < 0.05; 2 symbols if *p* < 0.01, and 3 symbols if *p* < 0.001.

CD4^+^ T cells from tolerant (Figure 4A) or naïve rats (Figure 3B) had similar proliferation to specific-donor PVG and third-party Lewis, and the response to self-DA was less than to either alloantigen. The similarity of the response of unfractionated and CD4^+^ T cells from tolerant hosts to PVG and third party was confirmed in limiting dilution assays (Figure 4B). This showed there is not full clonal deletion of CD4^+^ T cells reactive to PVG, as previously described in this model of tolerance (14).

### Comparison of the Response of CD4^+^CD25^−^ T Cells from Naïve and Tolerant Hosts in MLC

Removal of CD4^+^CD25^+^ T cells from naïve CD4^+^ T cells resulted in enhanced proliferation of the remaining CD4^+^CD25^−^ T cell population in MLC to PVG, Lewis, and self-DA, compared to that of unfractionated lymphoid cells (Figure 3A) and CD4^+^ T cells (Figures 3A,B). This suppression is not antigen-specific as the response to PVG, Lewis, as well as to self-DA was enhanced by removal of CD4^+^CD25^+^ T cells (Figures 3A,B) (29). The removal of naïve CD4^+^CD25^+^ T cells unmasked a significant autologous response in MLC, which was delayed compared to that to alloantigen, but at its peak was nearly as great as to alloantigen. This autologous response of naïve CD4^+^CD25^−^ T cells has been described previously, for example (29). The enhanced response by removal of naïve CD4^+^CD25^+^ T cells was confirmed in a serial dilution MLC, where the stimulator cells numbers were constant, and the responder cells were serially diluted twofold starting at 2 × 10^5^ cells per well, and out to 6.25 × 10^4^ per well (Figure 3C). There was significantly enhanced proliferation of naïve CD4^+^CD25^−^ T cells compared to unfractionated CD4^+^ T cells at several dilutions. Thus, with naïve hosts, the minority CD4^+^CD25^+^ T cells (10%) suppressed proliferation of the majority CD4^+^CD25^−^ T cells (90%) population within CD4^+^ T cells.

In contrast, CD4^+^CD25^−^ T cells from tolerant hosts had no increase in proliferation to specific-donor PVG compared to CD4^+^ T cells from tolerant hosts but had significantly greater proliferation to the third-party Lewis (Figure 4B). The reduced response of CD4^+^CD25^−^ T cells from tolerant hosts to PVG may be due to clonal pruning. This finding also suggested that the CD4^+^CD25^+^ T cells in unfractionated CD4^+^ T cells from tolerant hosts did not suppress proliferation of CD4^+^CD25^−^ T cells from tolerant hosts to specific-donor but inhibited these cells’ proliferation to third-party or self.

### Comparison of the Response of CD4^+^CD25^+^ T Cells from Naïve and Tolerant Hosts in MLC

There was a small and similar response of naïve CD4^+^CD25^+^ T cells to PVG or Lewis alloantigen that was greater than to self-DA (Figure 3B). This response to alloantigen peaked at day 3 and waned after day 4 (Figure 3A). Thus, we report day 3 proliferation.

The response of CD4^+^CD25^+^ T cells from tolerant animals was different. CD4^+^CD25^+^ T cells from tolerant hosts had significantly lower proliferation to PVG than to third party Lewis (Figure 4A). No increase in proliferation of CD4^+^CD25^+^ T cells from tolerant hosts to PVG alloantigen was observed on days 2 through to 5 (data not shown). Figure 4A, in the bottom row, shows results from four other separate experiments demonstrating low proliferation of CD4^+^CD25^+^ T cells from tolerant hosts to PVG that was not significantly different to the response to self-DA in most experiments. CD4^+^CD25^+^ T cells from tolerant hosts retained MLC responsiveness to third-party Lewis stimulator cells, similar to that of naïve CD4^+^CD25^+^ T cells.

This was a paradoxical finding, as it was assumed that donor alloantigen-specific CD4^+^CD25^+^ T cells would be increased in tolerant hosts.

### Examination of Cytokine Effect on Proliferation of CD4^+^CD25^+^ T Cells from Tolerant Hosts to Specific-Donor Alloantigen

We have shown that the survival *in vitro* of tolerance transferring CD4^+^ T cells requires both stimulation with specific-donor alloantigen and cytokines from activated lymphocytes (16, 18, 23, 24). Thus, we examined which T cell cytokines supported proliferation of CD4^+^CD25^+^ T cells from tolerant hosts to specific-donor antigen but not to third-party antigen or self-DA.

**Figure 5:**
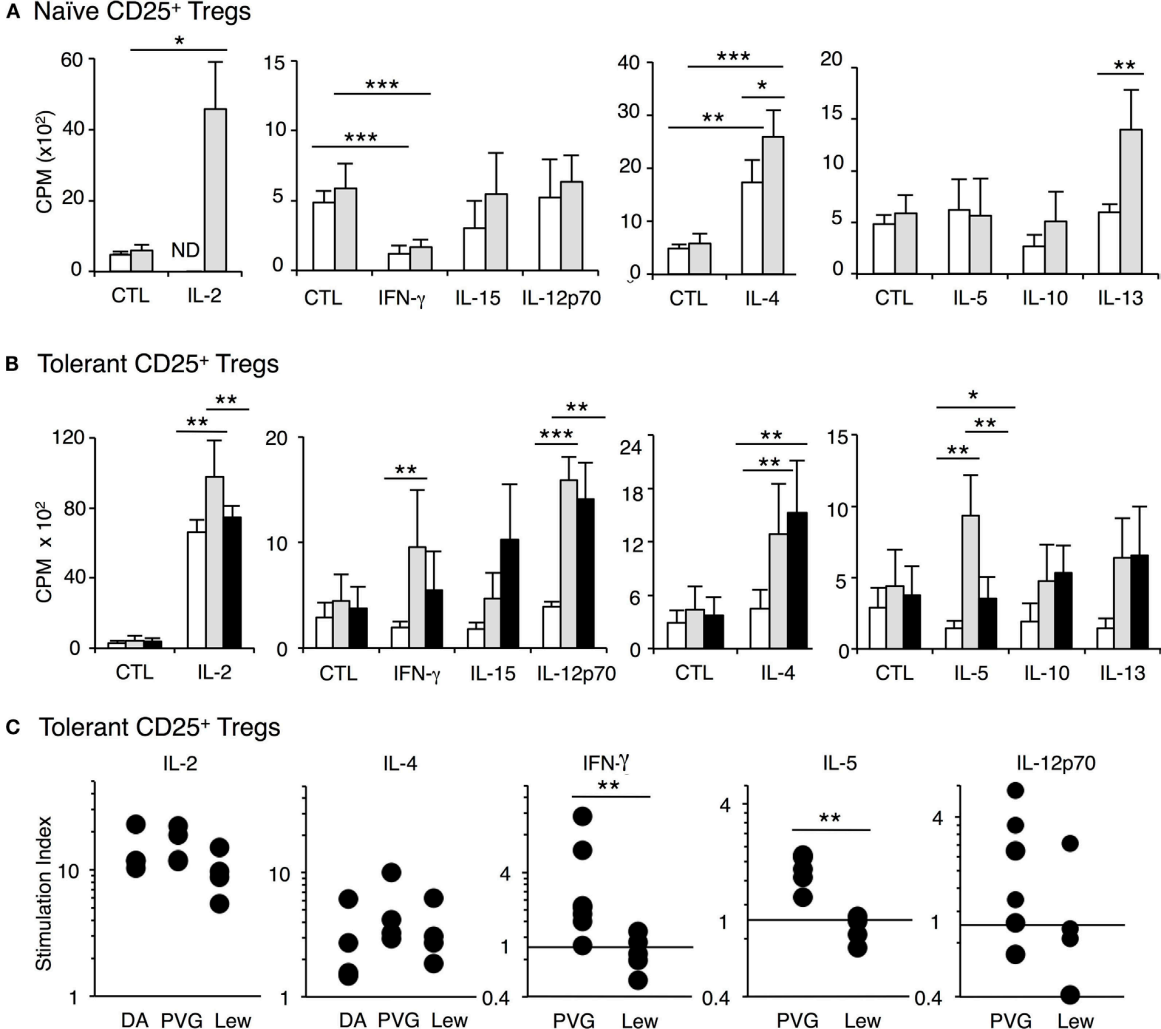
Examination of the effect of cytokines on proliferation of CD4^+^CD25^+^ T cells from tolerant and naïve hosts in mixed lymphocyte culture (MLC). Proliferation was assayed at 4 days when the effects of cytokines are maximal and proliferation of naïve CD4^+^CD25^+^ T cells is waning. Significant difference indicated as *p < 0.05, **p < 0.01, and ***p < 0.001. **(A)** Proliferation of naïve CD4^+^CD25^+^ T cells to self-DA (◻) and allogeneic Piebald Virol Glaxo rat strain (PVG) (◼) stimulator cells with cytokines. rIL-2 significantly (*p* < 0.05) enhanced the proliferation against PVG stimulators. rIL-4 enhanced proliferation to both self-DA (*p* < 0.01) or allogeneic donor PVG (*p* < 0.001) and the proliferation to PVG was significantly higher (*p* < 0.05). rIFN-γ inhibited naïve CD4^+^CD25^+^ T cell proliferation to self and PVG (*p* < 0.001). rIL-15, rIL-12p70, rIL-5, rIL-10 did not induce any proliferation of naïve CD4^+^CD25^+^ T cells to self or allogeneic PVG stimulator cells. In this experiment, but not in others, rIL-13 induced some proliferation to PVG (*p* < 0.01). These findings were replicated in two other separate experiments. **(B)** Proliferation of CD4^+^CD25^+^ T cells from tolerant hosts to self-DA (◻), specific-donor PVG (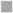) and third-party Lewis (◼) stimulator cells with cytokines. The key finding was that rIFN-γ and rIL-5 consistently induced increased proliferation of CD4^+^CD25^+^ T cells from tolerant hosts to specific-donor PVG and not to third-party or self (*p* < 0.01). rIL-12 p70 had variable effects. Both rIL-2 and rIL-4 markedly enhanced proliferation to self-DA, specific-donor PVG, and third-party Lewis. Although the proliferation with rIL-2 in this experiment was greater to PVG than to Lewis, this was not a consistent finding, as shown in Figure 5C. rIL-15, rIL-10, and rIL-13 did not induce proliferation to specific donor in any of the six separate experiments. **(C)** Stimulation index of CD4^+^CD25^+^ T cells from tolerant hosts in MLC with cytokines. The stimulation index for proliferation to specific-donor PVG was greater when rIFN-γ (**p < 0.01) (*n* = 7 and 5) or rIL-5 was added compared to that for third-party Lewis (**p < 0.01) (*n* = 6 and 4). rIL-2 or rIL-4 induced proliferation to self, PVG, and Lewis, and this effect was not antigen specific. The effect of rIL-12p70 was inconsistent but was increased to specific donor but not third party or self in 4 of 6 experiments.

Proliferation of naïve CD4^+^CD25^+^ T cells to all stimulator cells is enhanced by addition of rIL-2 or rIL-4 as previously described (22, 25, 26) and replicated in Figure 5A. rIL-2 and rIL-4 also induced proliferation of CD4^+^CD25^+^ T cells from tolerant hosts to self- or PVG and Lewis stimulator cells (Figure 5B). This polyclonal expansion by rIL-2 or rIL-4 was observed in four separate experiments. Neither rIL-2 nor rIL-4 selectively expanded CD4^+^CD25^+^ T cells from tolerant hosts to specific-donor PVG. The increased proliferation induced by rIL-2 or IL-4 to PVG and to third-party Lewis varied. Although there is a difference in the experiment in Figure 5B, this was not consistent, as stimulation index showed no difference in response to specific donor PVG and third party Lewis (Figure 5C).

Other Th1 cytokines, rIFN-γ, rIL-12p70, and rIL-15 did not enhance proliferation of naïve CD4^+^CD25^+^ T cells PVG, Lewis, or self-DA. In some cultures, rIFN-γ significantly suppressed proliferation (Figure 5A). The Th2 cytokines, rIL-5, rIL-10, and rIL-13 also did not enhance proliferation of naïve CD4^+^CD25^+^ Treg (Figure 5A). Thus, naïve CD4^+^CD25^+^ T cells were not activated by the other Th1 and Th2 cytokines we tested.

With CD4^+^CD25^+^ T cells from tolerant hosts, rIL-5 or rIFN-γ enhanced the response to specific-donor PVG but not to third-party Lewis or self-DA stimulator cells (Figure 5B).

With rIFN-γ, proliferation of CD4^+^CD25^+^ T cells from tolerant hosts was significantly enhanced to donor PVG compared to the response with rIFN-γ and DA stimulator cells, in 6 of 7 separate experiments (Figure 5C). The response to PVG with rIFN-γ was also greater than the response of CD4^+^CD25^+^ T cells from tolerant hosts to PVG where there was no cytokine or supernatant from non-transfected CHO-s cells (*p* < 0.01). rIFN-γ did not enhance proliferation of CD4^+^CD25^+^ T cells from tolerant hosts to third-party Lewis (*p* < 0.01) (Figure 5C) in any of five separate experiments. With rIFN-γ, the response to PVG was greater than the response to Lewis in all five separate experiments where there was direct comparison (*p* < 0.05–0.001). The reproducibility of this selective enhancement of proliferation by rIFN-γ to specific donor alloantigen is summarized as stimulation index in Figure 5C. With rIFN-γ, this response to specific donor is significantly different to proliferation to third-party, which was not enhanced by rIFN-γ (*p* < 0.01) (Figure 5C).

rIL-5 was the only Th2 cytokine that enhanced proliferation of CD4^+^CD25^+^ T cells from tolerant hosts to specific-donor PVG but not to self-DA or third party Lewis. This increased stimulation induced to PVG by rIL-5 was replicated in all six experiments (*p* < 0.05–0.01). No increased response to third party Lewis was observed in four experiments (*p* < 0.01). Comparing the effects of rIL-5 on proliferation expressed as stimulation indexes, the proliferation to specific-donor PVG was significantly greater than its effect on proliferation to third-party Lewis (Figure 5C).

rIL-12p70 enhanced proliferation of CD4^+^CD25^+^ T cells from tolerant hosts (Figure 4B), but this was not consistently observed (Figure 5C). 4 of 6 replicate experiments showed rIL-12 enhanced proliferation to PVG, and 1 of 4 showed enhanced proliferation to third party Lewis (Figure 5C). Comparison of stimulation indexes with rIL-12 showed no significant differences in responses to PVG and Lewis.

No other cytokine including rIL-15, rIL-12p40 (data not shown), rIL-10, rIL-13, nor rTGF-β (data not shown) enhanced proliferation of CD4^+^CD25^+^ T cells from tolerant hosts to either PVG or third-party Lewis alloantigen (Figure 5B).

### Comparison of the Cytokine Receptor Expression on CD4^+^CD25^+^ T Cells from Naïve and Tolerant Hosts Using RT-PCR

**Figure 6:**
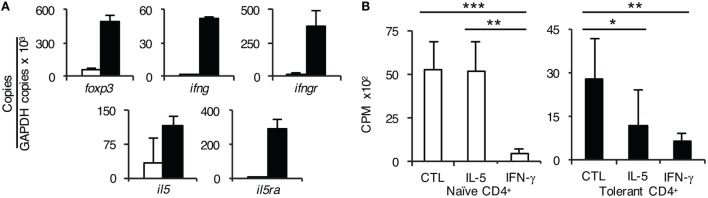
Further studies on the role of interferon (IFN)-γ and IL-5 on CD4^+^ T cells from tolerant hosts. **(A)** Expression of cytokine, cytokine receptors, and forkhead box P3 (Foxp3) on CD4^+^CD25^+^ T cells from naïve and tolerant rats was assessed by reverse transcription polymerase chain reaction of enriched CD4^+^CD25^+^ T cells from naïve (□) and tolerant (■) DA rats. CD4^+^CD25^+^ T cells from both naïve and tolerant hosts expressed *foxp3*, and those from tolerant hosts had higher expression of *foxp3*. CD4^+^CD25^+^ T cells from tolerant hosts but not naive cells expressed mRNA for *ifngr* and *il5ra*. Representative results of three experiments are presented. *ifng* and *il5* expression was also increased in CD4^+^CD25^+^ T cells from tolerant hosts, consistent with induction of Ts1 or Ts2 cells and/or Th1-like and Th2-like T regulatory cell (Treg), respectively, see Figure 7. **(B)** Effect of rIFN-γ and rIL-5 on proliferation to Piebald Virol Glaxo rat strain (PVG) in mixed lymphocyte culture (MLC) of CD4^+^ T cells from naïve and tolerant rats. To examine if the failure of CD4^+^CD25^+^ Treg from tolerant hosts to suppress CD4^+^CD25^−^ T cells in MLC was due to lack of a required cytokine; IFN-γ or IL-5 was added to MLC with unfractionated CD4^+^ T cells that had the natural mixture of both CD4^+^CD25^+^ and CD4^+^CD25^−^ T cells. With naïve CD4^+^ T cells (□), rIL-5 had no effect but rIFN-γ suppressed proliferation (*p* < 0.001). This is consistent with IFN-γ having non-specific antiproliferative effects, as previously described in rat MLC (29). With CD4^+^ T cells from tolerant hosts (■), rIL-5 inhibited the response to specific-donor PVG (*p* < 0.05) demonstrating suppression of CD4^+^CD25^−^ T cells by CD4^+^CD25^+^ Treg. rIFN-γ again was inhibitory (*p* < 0.01). Similar results were obtained in two other experiments.

**Figure 7:**
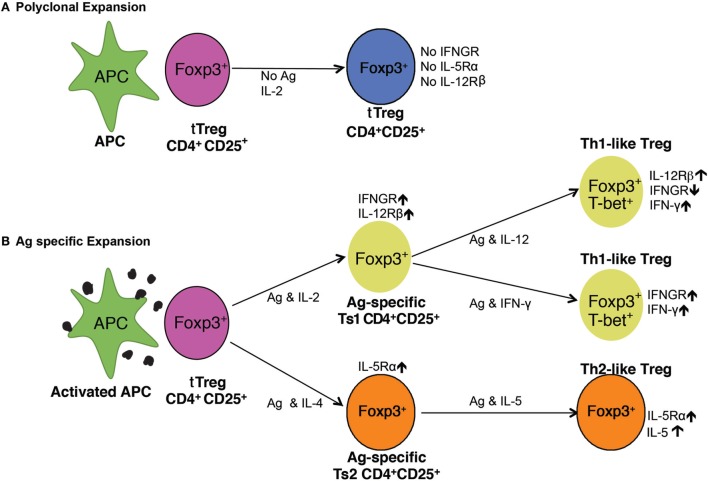
Pathways of activation of naïve CD4^+^CD25^+^ T regulatory cell (Treg) (tTreg). **(A)** Polyclonal expansion of tTreg. The most common expansion pathway of tTreg by IL-2 with non-antigen-specific stimulation leads to polyclonal expansion of tTreg, which do not express receptors for interferon (IFN)-γ, IL-5, or IL-12 and have no increased capacity to suppress. To suppress normal immune responses, tTreg are required at ratios of ≥1:1 (21, 29). **(B)** Antigen-specific expansion of tTreg. tTreg that have TCR for specific-alloantigen can be activated by antigen and either IL-2 or IL-4. tTreg with TCR for specific-alloantigen are activated by antigen and IL-2 to Ts1 cells, which have increased potency to suppress responses to specific-alloantigen (22). These cells are induced to express IFNGR and IL-12Rβ2 and can be further activated by either IFN-γ or IL-12 (25), in the presence of specific-alloantigen. This further activation is blocked if IL-2 is present (25). They are induced to express *T*-bet and IFN-γ, and are Th1-like Treg (25). These Th1-activated tTreg depend on IFN-γ or IL-12p70 as well as stimulation with specific-alloantigen for their survival and function. In the Th2 activation pathway, IL-4 and alloantigen induce Ts2 cells that express the specific receptor for IL-5Rα (22). IL-5 promotes Ts2 cells to become Th2-like Treg. IL-4 also, in the absence of antigen, induces polyclonal expansion of tTreg. These separate pathways of activation of tTreg by Th1 and Th2 cytokines explain the *in vitro* findings with CD4^+^CD25^+^ T cells from tolerant rats described here. The requirement of either IFN-γ or IL-5, and possibly other cytokine, for antigen-specific CD4^+^CD25^+^ Treg to survive, explains the findings that CD4^+^CD25^+^ T cells from tolerant hosts die *in vitro* and do not suppress unless key cytokines are present.

CD4^+^CD25^+^ T cells from tolerant hosts expressed *ifngr* and *il5ra* whereas naïve CD4^+^CD25^+^ T cells did not express *il5ra* and had low expression of *ifngr* (Figure 6A). Further, CD4^+^CD25^+^ T cells from tolerant hosts also expressed *il5* and *ifng*. These findings were consistent with our previous report of two pathways of alloactivation of naïve tTreg (22, 26), summarized in Figure 7.

CD4^+^CD25^+^ T cells from tolerant hosts had greater *foxp3* expression compared to those from naïve animals, consistent with activated Treg.

### Effect of Addition of rIFN-γ or rIL-5 to Cultures of CD4^+^ T Cells from Tolerant Hosts to Specific-Donor Alloantigen in MLC

To confirm the functional requirement of receptors for IL-5 and IFN-γ, we tested their effect on MLC of CD4^+^ T cells from tolerant hosts to PVG. As described above, CD4^+^ T cells from tolerant hosts had no enhancement in proliferation when the CD4^+^CD25^+^ T cells were removed, suggesting the CD4^+^CD25^+^ T cells from hosts did not suppress CD4^+^CD25^−^ T cells from tolerant hosts. We examined if this failure to suppress was due to insufficient IFN-γ or IL-5 in MLC. Both cytokines reduced proliferation of CD4^+^ T cells from tolerant hosts to specific-donor alloantigen in MLC compared to MLC without cytokines (Figure 6B).

However, rIFN-γ, but not rIL-5, also suppressed MLC of naïve CD4^+^ T cells (Figure 6). This effect of rIFN-γ is consistent with the known effect of IFN-γ on T cell proliferation (29). These results suggested IL-5 and possibly IFN-γ preserved the suppressor function of the antigen-specific CD4^+^ Treg in CD4^+^ T cells from tolerant hosts during stimulation in MLC.

## Discussion

CD4^+^CD25^+^FOXP3^+^ T cells suppress rejection and play a major role in maintenance of alloantigen-specific tolerance (18, 19, 20, 31, 32). Our key finding was that *ex vivo*, CD4^+^CD25^+^ T cells from tolerant animals did not proliferate to specific-donor alloantigen but retain the capacity to proliferate to third-party. This was a paradox, as it would be expected that this population would have an enhanced response to specific-donor stimulation in MLC.

This paradox can be explained by our finding that activated antigen-specific CD4^+^ T cells that can transfer alloantigen-specific tolerance rapidly die *ex vivo* (16, 23, 24) unless stimulated with specific-donor alloantigen and cytokines in supernatant from ConA-activated spleen cells (23, 24, 33). This suggests that the alloantigen-specific Treg need constant stimulation by specific-donor alloantigen and cytokines from an ongoing effector T cell response to the allograft. We have shown that rIL-2 (23) or rIL-4 (24) alone do not support survival of alloantigen-specific CD4^+^ Treg. Both IL-2 and IL-4 induced non-alloantigen-specific proliferation of naïve and tolerant CD4^+^CD25^+^ T cells. Thus, IL-2 or IL-4 did not distinguish between naïve and tolerant CD4^+^CD25^+^ T cells.

The cytokines required to maintain antigen-specific Treg were suggested in our studies on activation of naïve CD4^+^CD25^+^FOXP3^+^ T cell by alloantigen. rIL-2 induced expression of receptors for the Th1 cytokines IFN-γ (22) and IL-12 (25) but not Th2 cytokines IL-4 or IL-5 (22). rIL-4 induced expression of receptors for the Th2 cytokine IL-5 (22) but not for IFN-γ or IL-12. The two pathways for alloactivation of naïve tTreg by Th1 or Th2 cytokines are illustrated in Figure 7.

Here, we found CD4^+^CD25^+^ T cells from tolerant but not naïve hosts, expressed *ifngr* and *il5ra*. Further, CD4^+^CD25^+^ T cells from tolerant hosts in the presence of IFN-γ or IL-5 proliferated in MLC to specific-donor but not to self or third-party. Addition of rIL-5 to MLC with CD4^+^ T cells from tolerant, but not naïve animals, inhibited their proliferation to specific-donor. This suggested the suppressor function of the CD4^+^CD25^+^FOXP3^+^ T cells was maintained by IL-5. A direct effect on CD4^+^CD25^−^ T cells from tolerant hosts is unlikely as IL-5Rα is only expressed on some activated CD4^+^CD25^+^ Treg and not other CD4^+^ T cells (22). rIFN-γ suppresses naive CD4^+^ T cells’ proliferation in MLC (29) and, in this study, it suppressed proliferation of CD4^+^ T cells from tolerant hosts. The effects of rIFN-γ and rIL-5 on proliferation of alloantigen-specific CD4^+^CD25^+^ Treg showed that they expressed functional receptors for IFN-γ and IL-5.

CD4^+^CD25^+^ T cells from tolerant hosts had increased expression of *ifng* and *il5*. The expression of *ifng* was consistent with presence of Th1-like Treg, whereas the expression of *il5* was consistent with induction of Th2-like Treg (22) as illustrated in Figure 7.

rIFN-γ in MLC promotes generation of alloantigen-specific Treg (34, 35). IFN-γ also plays a role in regulation of autoimmunity (36, 37) that is mediated by CD4^+^CD25^+^ Treg. IFN-γ also promotes induction of Th1-like Treg (38, 39).

We have shown that rIL-5 promotes tolerance and activates *il5ra* expressing Treg that suppress Th1 responses in autoimmunity (26) and reduces induction of Th1 responses to allografts (40).

These results are consistent with alloantigen-specific CD4^+^CD25^+^ Treg from tolerant hosts being dependent upon IFN-γ or IL-5, and possibly other cytokines such as IL-12 (25) to maintain their suppressor function (Figure 7). Although we did not find a consistent effect of rIL-12 on proliferation of CD4^+^CD25^+^ T cells from tolerant hosts, this cytokine did induce proliferation in most experiments. rIL-12 also promotes activation of Th1-like Treg (25, 41) that prevents allograft rejection (25).

The failure of CD4^+^CD25^+^ T cells from tolerant hosts to proliferate to specific-donor strain stimulator cells suggests there is depletion of naïve tTreg with TCR for specific-donor. This depletion of specific-donor reactive tTreg could be because all naïve tTreg with TCR reactive to specific-donor alloantigen have been activated leaving no naïve tTreg with TCR that recognizes donor alloantigen.

The finding that CD4^+^CD25^−^ T cells from tolerant hosts had reduced proliferation to specific donor, compared to naïve CD4^+^CD25^−^ T cells was consistent with some depletion or clonal pruning, that only manifest when CD4^+^CD25^+^ T cells are removed. The apparent normal reactivity of either unfractionated or CD4^+^ T cells from tolerant hosts to specific-donor was due to lack of suppression by CD4^+^CD25^+^ T cells. Reduced alloreactivity of CD4^+^CD25^−^ T cells from tolerant hosts to specific-donor, but not to third party, has been observed in human renal transplants with reduction in T cells with TCR reactive to specific-donor (8).

Clonal pruning does not explain tolerance as CD4^+^CD25^−^ T cells from tolerant rats effect rejection of specific-donor grafts (19) and very few CD4^+^ T cells (10, 18) or CD4^+^CD25^−^ T cells (21) are required to mediate fully allogeneic graft rejection. Tolerant hosts have sufficient donor reactive CD4^+^CD25^−^ T cells to effect rejection, but these are either not activated by the graft (42) or are suppressed by Treg (3, 10, 18, 19).

Relevant to humans, IL-4 and alloantigen activated human Treg express IL-5Rα (26) and Th1-like Treg are dependent upon IFN-γ in renal transplant patients (43).

Development of operational tolerance to an allograft, so that toxic non-specific immunosuppressive drugs can be reduced or removed, is a desired aim in clinical organ transplantation. Existing immunosuppressive drugs are associated with higher risks of infections, malignancy, vascular disease, and metabolic effects such as diabetes, osteoporosis, and renal impairment. Reliable tests to detect transplantation tolerance could provide a valuable tool to determine the ongoing need for immunosuppression.

Renal transplant patients with operational tolerance after immunosuppressive therapy has been stopped, have increased numbers of CD4^+^CD45RA^−^FOXP3^hi^ memory Treg, with increased expression of hypo-methylated Treg-specific demethylated region for FOXP3, and higher levels of CD39 and GITR (44), consistent with an expanded memory Treg population. Human memory Treg with low or no expression of CD45RA that express CD44 rapidly die *ex vivo* (45). Thus, human antigen-specific Treg may require specific-antigenic stimulation and cytokines to survive.

Gene expression in peripheral blood lymphocytes of liver and renal transplant patients on no immunosuppression compared to those with ongoing immunosuppression identified B cell signature in five studies (46–49). The same genes were not expressed in all studies but a combined a common set was found (50). A Treg signature was not identified.

These studies identified several characteristics of CD4^+^ T cells from tolerant hosts that could be used to diagnose alloantigen-specific transplant tolerance include (1) CD4^+^CD25^−^ T cells’ response to specific-donor is not greater than CD4^+^ T cells, whereas their response to third-party is greater than CD4^+^ T cells; (2) CD4^+^CD25^+^ T cells from tolerant hosts do not proliferate to specific-donor unless IFN-γ or IL-5 are present, but do proliferate to third-party without IFN-γ or IL-5.

## Ethics Statement

The study was carried out in accordance of the “Australian Code for the Care and Use of Animals for Scientific Purposes (NHMRC)” and Animal Ethics Committee of the University of New South Wales (UNSW), Australia. Animal experimental protocols were approved by the Animal Ethics Committee of UNSW Australia.

## Author Contributions

BH, SH, KP, NV, GT, and NC initiated and designed the research protocols and methods. CR, KP, NV, NC, GT, MN, and RB performed experiments. BH, CR, KP, NV, SH, GT, and RB analyzed the results. BH, NV, KP, SH, GT, and CR wrote the paper.

## Conflict of Interest Statement

BH and SH hold patents related to production of antigen-specific Treg and tests of tolerance related to this work. No author holds other commercial interests related to this work.

## Abbreviations

Ag, antigen; CD, cluster of differentiation; ConA, Concanavalin A; DA, dark agouti strain of rats; FOXP3, forkhead box P3; IFN, interferon; Lew, Lewis rat strain; MLC, mixed lymphocyte culture; PVG, Piebald Virol Glaxo rat strain; R, receptor; r, recombinant; Rt-1, rat MHC; RT-PCR, reverse transcription–polymerase chain reaction; Th, CD4 T helper cell; Treg, T regulatory cell; tTreg, thymic-derived Treg; TSDR, Treg-specific demethylated region.
